# Obesity-Related Skin Conditions: Exploring the Link

**DOI:** 10.7759/cureus.57772

**Published:** 2024-04-07

**Authors:** Guntamukkala Geeta Sai, Thillaikkarasi A, Afthab Jameela Wahab

**Affiliations:** 1 Department of Dermatology, Saveetha Medical College and Hospital, Saveetha Institute of Medical and Technical Sciences (SIMATS) Saveetha University, Chennai, IND

**Keywords:** acrochordons, acanthosis nigricans, body mass index, cutaneous manifestations, obesity

## Abstract

Background

Obesity is a medical condition characterized by the accumulation of excess fat that can negatively impact health, resulting in a decreased life expectancy and heightened health issues. Obese patients experience skin changes caused by skin infections, mechanical friction, and various skin hypertrophic conditions like fibromas and acanthosis nigricans. Approximately 60-70% of patients suffering from obesity exhibit a range of skin changes.

Objective

The main objective of the present study is to identify the various types of skin conditions linked to obesity and investigate their relationship with body mass index (BMI).

Methodology

This is a cross-sectional observational study. This study included obese patients with a BMI greater than 30 kg/m^2^ who visited the dermatology outpatient department at Saveetha Medical College and Hospital in Chennai, India. We enrolled 100 patients in this study. After obtaining consent, demographic information, height, weight, and cutaneous examination were conducted, and the results were documented. Statistical analysis was conducted using the chi-squared test, where P<0.05 was considered significant. The t-test for independent samples was done to analyze quantitative variables.

Results

The mean age at presentation was 39.3, and the standard deviation was 9.9. The average BMI was 34.3, and the standard deviation was 2.6. Of the total patients, 34% belonged to the 31-40-year age group, which was followed by 30% in the 41-50-year age group, 23% belonged to the 19-30-year age group, 11% belonged to the 51-60-year age group, and 2% belonged to the >60-year age group. Most patients (63%) had Class I obesity (BMI 30.00-34.99), 34% had Class II obesity (BMI 35.00-39.99), and 3% had Class III obesity (BMI >40.00). The most common cutaneous manifestation overall was acrochordons, followed by acanthosis nigricans, striae distensae, infections, and psoriasis.

Conclusion

Obesity is identified as a significant public health issue, and its association with skin problems is of practical importance for many clinicians.

## Introduction

Obesity is one of the known chronic metabolic diseases characterized by an excessive accumulation of abnormal body fat, leading to a noticeable increase in the overall weight of the body. It is multifactorial and has endocrine implications. Undoubtedly, obesity is to be considered a global public health concern of considerable scale, posing a challenge to healthcare systems due to its complicated management and the financial implications of its comorbidities.

Skin, being the largest organ of the body, performs various defensive functions and helps in maintaining homeostasis in mammals. Skin has long been considered a "mirror," reflecting the actual health status of various human body internal organs throughout history. In the past decades, the understanding of obesity has shifted from being viewed as an isolated condition to being recognized as one of the systemic diseases affecting multiple organs and systems [1]. The physiology and pathology of the skin are affected by the dynamic interplay of immune signaling molecules, hormones, and growth factors. As a result, skin homeostasis serves as a reflection of the inner state of the organism [2,3]. Obesity causes various changes in the body, such as impaired skin barrier function, decreased sebum production, changes in sweat gland function, impact on the lymphatic system, alterations in collagen structure and function, delayed wound healing, and effects on microcirculation, macrocirculation, and subcutaneous fat.

Obesity is related to a range of skin conditions, like psoriasis, acanthosis nigricans, keratosis pilaris, plantar hyperkeratosis, hirsutism, hidradenitis suppurativa, striae distensae, acrochordons, adiposis dolorosa, lymphedema, fat redistribution, chronic venous insufficiency, cellulitis, and skin infections [4].

Obesity is a prevalent issue in the 21st century, resulting in a notable rise in obese patients seeking dermatology outpatient department services. The diverse skin conditions associated with obesity are important for both dermatologists and physicians treating obesity. This study aimed to identify the different skin manifestations of obesity and examine their correlation with body mass index (BMI).

## Materials and methods

The study is a cross-sectional observational study. Our study included obese patients with a BMI greater than 30 kg/m^2^ who visited the dermatology outpatient department at Saveetha Medical College and Hospital in Chennai. This study included all patients over 18 years old with one or more specific skin conditions. Excluded individuals include pregnant women, lactating mothers, and immunocompromised patients. Following obtaining consent, demographic information, weight, height, and skin examination outcomes were documented. This study involved 100 patients of both genders. The grading of obesity was done according to the World Health Organization (WHO) classification (Table 1).

**Table 1:** WHO grading of obesity BMI: body mass index; WHO: World Health Organization

Weight status	BMI (kg/m^2 ^)
Underweight	<18.5
Normal range	18.5-24.9
Overweight	25-29.9
Obesity	≥30
Obese-Class I	30.0-34.9
Obese-Class II	35.0-39.9
Obese-Class III	≥40

## Results

**Figure 1:**
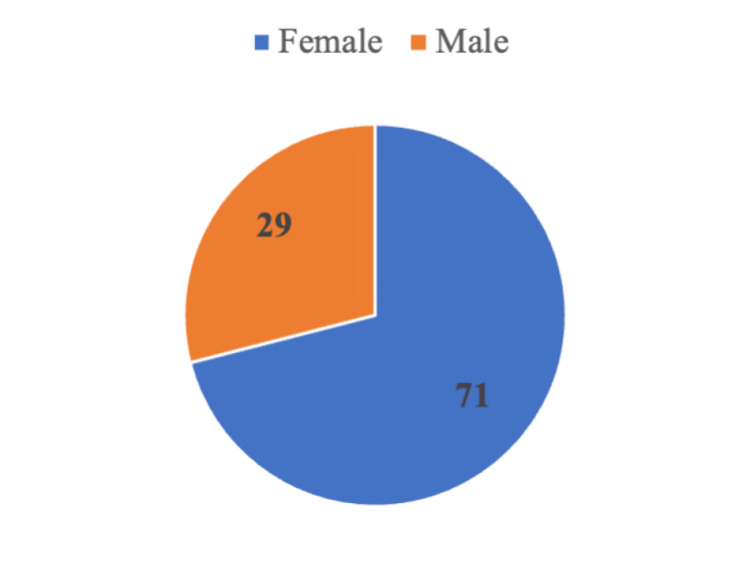
Sex-wise distribution of the participants

**Figure 2:**
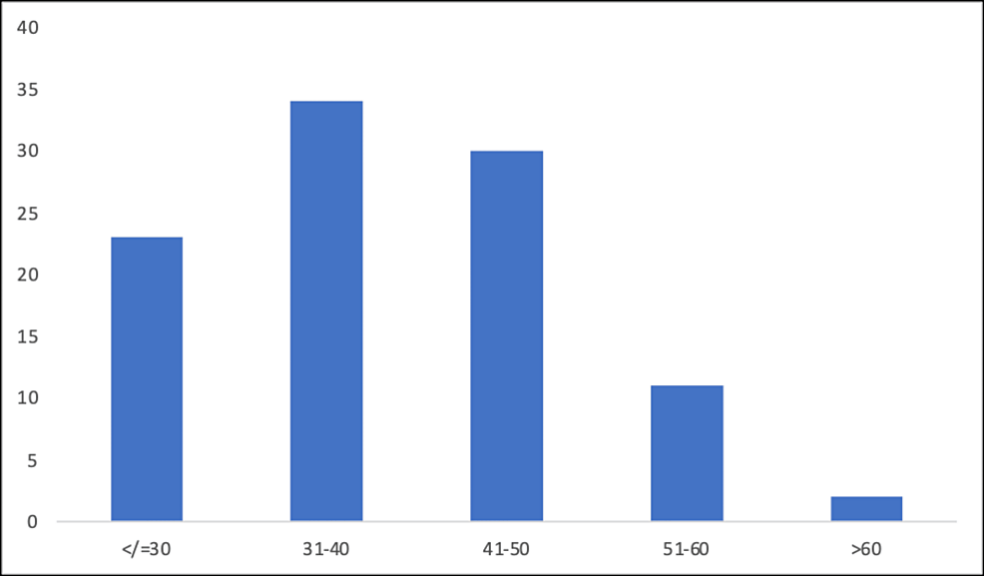
Age-wise distribution of the participants

Statistical analysis was conducted using the chi-squared test, where P<0.05 was considered significant. The t-test for independent samples was done to analyze quantitative variables. Analyzed were 100 patients, 71 of whom were female and 29 were male (Figure 1). The mean age at presentation was 39.3, and the standard deviation was 9.9. The age-wise distribution of participants is shown in Figure 2. The average BMI was 34.3, and the standard deviation was 2.6. Of the total patients, 34% belonged to the 31-40-year age group, which was followed by 30% in the 41-50-year age group, 23% belonged to the 19-30-year age group, 11% belonged to the 51-60-year age group, and 2% belonged to the >60-year age group. Most patients (63%) had Class I obesity (BMI 30.00-34.99), 34% had Class II obesity (BMI 35.00-39.99), and 3% had Class III obesity (BMI >40.00). The demographic data of the patients is shown in Table 2.

**Table 2:** Frequency distribution of variables BMI: body mass index

Variables	Frequency	Percentage
Sex		
Female	71	71
Male	29	29
Age		
19-30	23	23
31-40	34	34
41-50	30	30
51-60	11	11
>60	2	2
BMI		
30.0-34.9	63	63
35.0-39.9	34	34
>40	3	3

**Figure 3:**
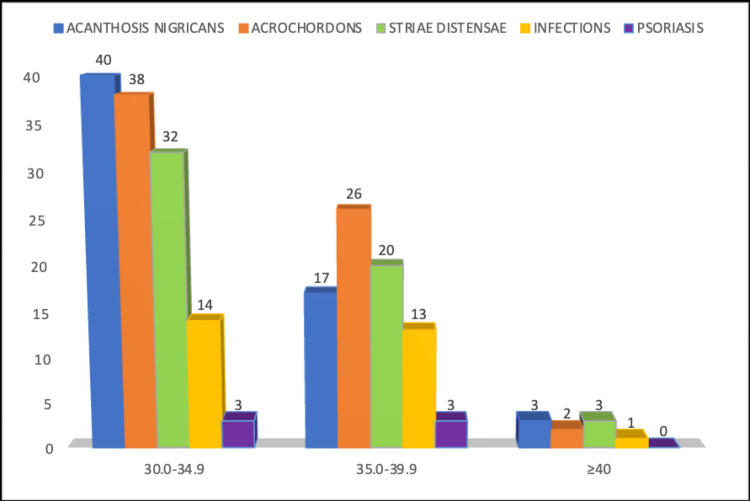
Distribution of cutaneous manifestations among obese patients according to BMI BMI: body mass index

Acanthosis nigricans, infections, acrochordons, psoriasis, and striae were among the several skin abnormalities observed in our study. The most common cutaneous manifestation overall was acrochordons, followed by acanthosis nigricans, striae distensae, infections, and psoriasis. The most common cutaneous manifestation among individuals with Class I obesity was acanthosis nigricans, followed by acrochordons, striae distensae, infections, and psoriasis (Figure 3). The association between gender and various cutaneous manifestations is shown in Table 3. Association with diabetes mellitus was observed in 46% and hypertension in 36% of the individuals.

**Table 3:** Association between sex and variables (* indicates statistically significant value)

Variables	Female	Male	P-value
Acanthosis nigricans	37 (61.7%)	23 (38.3%)	0.012*
Acrochordons	51 (77.3%)	15 (22.7%)	0.054
Striae distensae	40 (72.7%)	15 (27.3%)	0.674
Bacterial infections	1 (25.0%)	3 (75.0%)	0.198
Fungal infections	17 (73.9%)	6 (26.1%)	0.198
Viral infections	1 (100%)	0 (0%)	0.198
Psoriasis	6 (100%)	0 (0%)	0.106
Diabetes mellitus	36 (78.3%)	10 (21.7%)	0.14
Hypertension	27 (75.0%)	9 (25.0%)	0.509

## Discussion

Obesity is usually related to a variety of mucocutaneous manifestations [5,6]. In our study, the mean age at presentation was 39.3, and the standard deviation was 9.9. The average BMI was 34.3, and the standard deviation was 2.6. In this study, the frequency of dermatological conditions was higher in females. Insulin resistance, commonly encountered in obese patients [7], has been proven to cause specific changes in the skin. An association between hypertriglyceridemia, insulin resistance, BMI, and the occurrence of acrochordons has been reported. In our study, acrochordons were observed in 57.6%, 39.4%, and 3% of patients with BMI grades of 30.0-34.9, 35.0-39.9, and >40, respectively. Ahsan et al. [8] discovered a substantial relationship between acanthosis nigricans and diabetes mellitus. One of the most frequent conditions found in obese people is acanthosis nigricans. Acanthosis nigricans is linked to several comorbid illnesses in addition to obesity, including diabetes, endocrine disorders, metabolic syndrome, and hyperinsulinemia.

Hud et al. [9] found that higher plasma insulin levels and acanthosis nigricans were present in 74% of the obese population. In our study, we noted that acanthosis nigricans was observed in 66.7%, 28.3%, and 5% of patients with BMI grades of 30.0-34.9, 35.0-39.9, and >40, respectively.

Several studies showed that striae increased with a rise in the grades of BMI. Striae may develop due to heightened skin tension resulting from excessive weight gain [10]. According to Hsu et al. [11], children with moderate to severe obesity had a frequency of 40%, and the incidence increased with the longer duration of obesity. Cho et al. [12] reported a prevalence of 83.4% in 2006, with both obese and non-obese individuals included. In our study, we observed that about 58.2%, 36.4%, and 5.5% of patients presented with striae distensae in the BMI grades of 30.0-34.9, 35.0-39.9, and >40, respectively. The literature has highlighted the link between skin infections and obesity [6,13]. Increased skin surface area and friction due to rubbing lead to an increase in moisture and can accelerate the development of bacterial and fungal diseases. In this study, we noted fungal infections in 23% and bacterial infections in 4% of the patients. Boza et al. [14] conducted a comparative study in which they found that the following cutaneous manifestations had a statistically significant relationship with obesity when compared to the control group: intertrigo (P<0.001), pseudoacanthosis nigricans (P<0.001), lymphedema (P=0.002), striae (P<0.001), plantar hyperkeratosis (P<0.001), and acrochordons (P=0.007). Additionally, the presence of bacterial infections, striae, and pseudoacanthosis nigricans was found to correlate with the degree of obesity.

Psoriasis has been linked to several variables. Following the control for diabetes mellitus, dyslipidemia, and metabolic syndrome, the association with obesity was no more significant. It is recognized that psoriasis is a systemic illness linked to obesity, metabolic syndrome, dyslipidemia, hypertension, and type 2 diabetes. Additionally, there is evidence linking obesity to the development of psoriasis, and a relationship exists between BMI and the Psoriasis Area and Severity Index (PASI). Patients with psoriasis had a higher incidence and prevalence of obesity, according to Armstrong et al. [15]. Psoriasis vulgaris was discovered in 6% of the participants in our study. Obese people need to pay extra attention to skincare because they are more likely to develop treatable diseases and because their morbidity is higher than average. Skin conditions such as pseudoacanthosis nigricans and acrochordons are associated with endocrine disorders and should be investigated. Obese people have a higher risk of skin infections, which should be taken into consideration.

The limitations of the study were that the findings may not represent the broader population due to the relatively small sample size. In this study, we missed correlating the specific types of obesity with the specific types of skin diseases.

## Conclusions

Obesity is identified as a significant public health issue. The relationship between obesity and skin is crucial for clinicians, as understanding the complications of obesity can help reduce its negative health impacts.
